# Thromboelastography to Monitor Clotting/Bleeding Complications in Patients Treated with the Molecular Adsorbent Recirculating System

**DOI:** 10.1155/2011/313854

**Published:** 2011-03-06

**Authors:** Esther B. Bachli, Jörg Bösiger, Markus Béchir, John F. Stover, Reto Stocker, Marco Maggiorini, Eberhard L. Renner, Beat Müllhaupt, Reto A. Schuepbach

**Affiliations:** ^1^Medical Intensive Care Unit, University Hospital Zurich, 8091 Zurich, Switzerland; ^2^Clinic of Internal Medicine, Hospital Uster, 8610 Uster, Switzerland; ^3^Division of Haematology, University Hospital Zurich, 8091 Zurich, Switzerland; ^4^Surgical Intensive Care Unit, University Hospital Zurich, HOF-B-110, Raemistraße 100, 8091 Zurich, Switzerland; ^5^Division of Gastroenterology and Hepatology, University Hospital Zurich, 8091 Zurich, Switzerland; ^6^Multiorgan Transplant Program, University Health Network, University of Toronto, Toronto, Canada ON M5G 2N2

## Abstract

*Background.* The Molecular Adsorbent Recirculating System (MARS) has been shown to clear albumin-bound toxins from patients with liver failure but might cause bleeding complications potentially obscuring survival benefits. We hypothesized that monitoring clotting parameters and bed-side thromboelastography allows to reduce bleeding complications. *Methods*. Retrospective analysis of 25 MARS sessions during which clotting parameters were monitored by a standardized protocol. *Results*. During MARS therapy median INR increased significantly from 1.7 to 1.9 platelet count and fibrinogen content decreased significantly from 57 fL^−1^ to 42 fL^−1^ and 2.1 g/L to 1.5 g/L. Nine relevant complications occurred: the MARS system clotted 6 times 3 times we observed hemorrhages. Absent thrombocytopenia and elevated plasma fibrinogen predicted clotting of the MARS system (ROC 0.94 and 0.82). Fibrinolysis, detected by thromboelastography, uniquely predicted bleeding events. *Conclusion*. Bed-side thromboelastography and close monitoring of coagulation parameters can predict and, therefore, help prevent bleeding complications during MARS therapy.

## 1. Introduction

Due to limited donor liver availability, only selected patients with acute or acute-on-chronic liver failure profit from liver transplantation [1]. The majority of patients receive supportive therapy, and mortality remains high [2]. Various artificial liver support systems have been developed and proposed as bridging therapy until liver function recovers or a donor organ becomes available [3]. The Molecular Adsorbent Recirculating System (MARS), a cell-free albumin dialysis device, has been shown to remove albumin bound compounds and toxins and to exert a number of beneficial hemodynamic effects in patients with acute and acute-on-chronic liver failure [4]. However, to date, neither MARS nor any other liver support system has been unequivocally proven to alter clinical outcomes such as survival, a conclusion based on a meta-analysis [5] and yet unpublished multicenter clinical trials (HELIOS study, RELIEF trial; European Association for the Study of the Liver 2010).

Bleeding disorders are a major concern in all patients with liver failure [6]. MARS involves an extracorporeal blood circuit through a filter. This can lead to activation of platelets and the coagulation system [7]. Recent reports describe an association of severe and potentially fatal bleeding complications with MARS therapy [8–10]. 

 Patients' age, vasopressor therapy, pretreatment INR, fibrin D-dimer and fibrinogen concentrations [8], factor VIII, von Willebrand factor and severely altered thromboelastography parameters [9] were identified as risk factors for bleeding complications during MARS therapy. However, the predictive accuracy of each of these parameters was relatively low, limiting their use during individual MARS sessions in individual patients. 

Routine coagulation parameters are not useful in predicting bleeding complications in general. They cannot be used reliably to monitor coagulopathy during MARS due to the delay until the results are available at the bed-side. Thromboelastography is successfully used for managing coagulopathy during liver transplantation [11]. The role of bed-side monitoring of coagulation using thromboelastography during MARS has been discussed controversially [9, 10]. Although thromboelastography parameters were found to be altered by MARS therapy, the large variability of all parameter tested precluded the tests from accurately identifying patients at risk [9]. 

 In another study using thromboelastography to monitor patients receiving MARS no bleeding episodes occurred. Thus, its value in predicting complications could not be estimated [10]. 

 We hypothesized by combining thromboelastography and determination of standard clotting parameters, coagulopathy could be recognized very early during the MARS sessions. In patients receiving MARS we thus performed bed-side monitoring of coagulation using a standardized protocol for thromboelastography and determination of standard clotting parameters. This allowed to obtain coagulation parameters timely linked to the MARS sessions. We postulated that bleeding complications could thus be reduced by either stopping MARS and/or substituting coagulation products even before occurrence of bleeding complications. In this post hoc analysis we investigated if and which parameters might allow to identify complicated MARS sessions. 

## 2. Patients and Methods

### 2.1. Patients

At our institution, the largest tertiary hospital in Switzerland, patients with acute liver failure (ALF), acute-on-chronic liver failure (AoCLF), intractable pruritus of hepatic origin, or toxic drug accumulation because of impaired hepatic clearance are eligible for MARS treatment. A clinical protocol had been approved by the local Ethics Committee. Potential candidates were identified by the primary physician and were then evaluated for initiation of MARS therapy by an interdisciplinary team consisting of intensive care physicians (R. A. Schuepbach, R. Stocker, M. Béchir, E. B. Bachli) and hepatologists (B. Müllhaupt, E. L. Renner). After approval for MARS therapy by the interdisciplinary team, informed consent was obtained from all patients (or their next of kin). The present trial was approved by the local Ethics Committee, and conducted in accordance to the declaration of Helsinki.

### 2.2. Data Collection

Twenty five MARS sessions performed in 5 patients at the University Hospital Zurich, Switzerland, were retrospectively analyzed. Data on coagulation and thrombelastography had been prospectively collected according to a protocol specified below. The protocol was formulated by the above-mentioned physicians with the aim of preventing complications in patients receiving MARS. 

Data was retrospectively extracted from (i) the electronic hospital data base (KISIM, Cistec, Switzerland) capturing laboratory and clinical data, (ii) from the RoTeg thromboelastography analyzer and (iii) the paper chart of each patient containing all nursing notes and hand written ICU flow sheets which included demographics, diagnosis of underlying liver disease, indication for MARS therapy, time/duration of MARS sessions, pretreatment Child-Pugh [12], MELD [13], and SAPS II [14] sores, mechanical ventilation and/or vasopressor support, pre, on- and post-treatment laboratory parameters, time (relative to MARS), quantity and entity of transfusions and concomitant administration of medications eventually interfering with coagulation and/or clotting tests such as heparin. 

The following outcome data was extracted, change in clotting parameters between a baseline sample collected immediately prior to starting MARS and a sample drawn after one hour of MARS and at the end of the MARS, overt bleeding during MARS from any site, requirement/amount of transfusions of red cells, platelets and coagulation products (FFP, fibrinogen, clotting factors), duration of MARS sessions and cause of premature termination, as well as death within 30 days of MARS treatment.

### 2.3. MARS Dialysis Therapy

The Molecular Adsorbent Recircultating System (MARS; Teraklin AG, Rostock, Germany) was utilized according to the manufacturer's instructions. The system was connected to a PRISMA (Gambro Hospal, Glattbrugg, Switzerland) hemofilter pump. The extracorporeal blood circuit was primed with unfractionated heparin (5000 IU) during filling and thereafter flushed with 0.9% NaCl. Then MARS was connected to a large double lumen dialysis catheter in either subclavian or jugular position. Blood flow was set at 120 mL/min, flow within the albumin circuit of the MARS system at 150 mL/min, dialysate flow at 33 mL/min and net fluid balance at zero. In patients in whom bleeding risk was felt to be low or who had thrombotic problems with extracorporeal devices such as hemofilters either 400 IU/h of unfractionated heparin or Flolan (epoprostenol sodium; GlaxoSmithKline, Münchenbuchsee, CH) was infused directly into the extracorporeal circuit (immediately prior to the MARS filter). If tolerated and feasible, at least three initial MARS sessions of up to 10 hours duration were performed (Table 1). Continuation depended on the laboratory and clinical evolution of the patient, as judged by the interdisciplinary team and the primary responsible physicians.

### 2.4. Laboratory Methods and Standardisation of Thromboelastography

As a consequence of our previous observation study [8] clotting parameters were closely monitored in all patients receiving MARS using a standardized protocol. Thus, venous blood was collected from all patients immediately before, and 1, 2, and 4 hours after the start, as well as after completion of each MARS session using EDTA, heparin and plain vacutainer tubes (Becton-Dickinson, San Jose, CA, USA), as required for the different analysis. Samples were analyzed within 2 hours of collection. Blood counts, prothrombin time (PT with international standardize ratio INR), activated partial thromboplastin time, (aPTT), thrombin time (TT), fibrinogen, D-dimer, bilirubin, creatinin, bilirubin, and C reactive protein (CRP) were determined in the hospital's accredited laboratory using standard methods.

Bed-side thromboelastography was performed on a semi automated device (RoTeg, Pentafarm, Germany) according to the manufactures instructions. Blood samples were collected in EDTA containing vacutainer tubes (Becton-Dickinson, San Jose, CA, USA). Using the automated pipette of the RoTeg-device, samples of 300 *μ*L whole blood were recalcificated by StarTeg (Calciumcholride by Pentapharm, Germany) after adding InTeg (Pentafarm, Germany), an aPTT-like activator.

**Figure 1:**
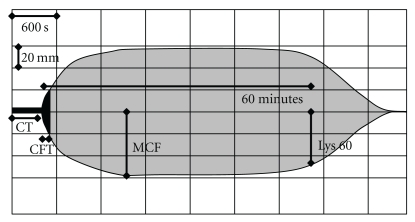
Scheme of parameters obtained by thromboelastography: Given are the clotting time (CT), the time in seconds passing until initial clot formation (CFT), the time in seconds passing until a strength of the clot of 20 mm is reached, the maximal clot firmness (MCF), an arbitrary unit in mm which reflects the maximal clot strength representing the interaction of fibrin and platelets and the remaining relative clot firmness after 60 minutes (Lys 60; %), representing fibrinolysis. Time is presented on the *x*-axis (horizontal; grid 600 seconds), clot strength on the *y*-axis (vertical; grid 20, resp., 40 mm).

To establish reference values for thromboelastography we relied on 40 volunteers who had signed an informed consent. Data was analyzed from 20 men and 20 women (aged 24 to 55 years). All volunteers had completed a standardised health questionnaire and all laboratory parameters (INR, aPTT, TT, fibrinogen, D-dimer, platelets, leucocytes, hemoglobin, creatinin, bilirubion, CRP and ferritin) were within normal limits. In each volunteer thromboelastography was performed in duplicate and if inter-individual differences exceeded 5%, the analysis was repeated. Normal range of parameters was defined as values between the 2.5 and the 97.5 percentile. Normal ranges were established for the clotting time (CT; i.e., the time in seconds between adding calcium to the blood sample and detection of clot formation to begin), clot firmness (CMF; i.e., an historical arbitrary unit in millimetres reflecting the clot strength; basing on the impedance of pin movement), clot formation time (CFT; i.e., the time that had passed (in seconds) between detecting of clot formation to begin and achievement of a clot strength with 20 mm amplitude), and the relative firmness of the clot after 60 minutes in relation to the maximal firmness (in %; Lys 60′) (Figure 1). Normal ranges of each parameter are summarized in Table 2 and are consistent with the values given by the manufacture. 

 In a subset of patients frozen plasma samples (stored at −80°C) were analyzed for prothrombin fragments (Enzygnost* F_1+2_, Dade Behring Marburg GmbH, Germany) and the endogenous calibrated thrombin potential (ETP) was determined using the Thrombinoscope Package (software, reagents, fluorometer; ThrombinoScope; Maastricht, NL). Measurements were done according to the manufacturer's instructions [15].

### 2.5. Missing Values

Analysis for each clotting parameter and each session were performed if at least one parameter was available from before starting MARS treatment (up to 6 hours before MARS start), a second obtained during a time window of 30 to 60 minutes after MARS was started and a third obtained in a time window of 0 to 6 hours after MARS was discontinued. During two uneventful sessions (patient no. 2) thromboelastography was not performed as a deviation from the standard operating procedure.

### 2.6. Data Analysis and Statistics

Data are shown as line plots (dots present individual data pairs linked by lines), box and whisker plots (with the box containing the middle 50% of the data and being divided up by the line representing the median, whiskers extending to 1.5 times the interquartile range above and below the 75th and 25th percentile, and closed dots representing outliers), or dot plots (mean ± standard deviation). For comparisons of two groups of either paired or unpaired samples we used the nonparametric Wilcoxon analysis and for 3 sample comparison the nonparametric Kruskal-Wallis one-way ANOVA on ranks. If the ANOVA analysis was significant, Bonferroni pair-wise group comparison was performed. Statistical analyses and data editing and presentation were performed using NCSS (NCSS 2007; Kaysville, Utah) and SPSS (SPSS Statistics 17.0; Chicago, Illinois) software packages.

## 3. Results

### 3.1. Patients and Baseline Characteristics

Baseline characteristics of the 5 patients undergoing a total of 25 MARS sessions are summarized in Table 3. Three of the five patients suffered from a lowered level of consciousness due to markedly impaired liver function. They required intubation for airway protection, were mechanically ventilated and required intermittent vasopressor support during the majority of the MARS sessions. Three of the five patients died within 30 days of MARS therapy. The 2 other patients (the patient with primary biliary cirrhosis and the patient with severe intoxication) survived more than 90 days after MARS therapy.

### 3.2. Efficacy of MARS Treatment

**Figure 2:**
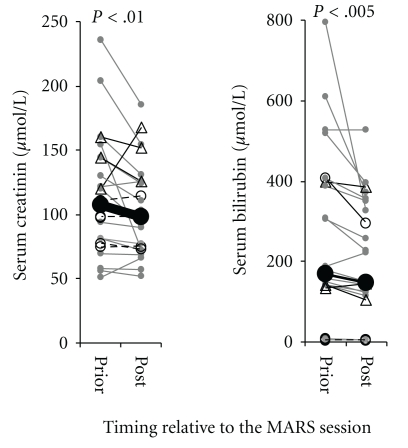
MARS therapy efficiently clears serum creatinin and bilirubin. Evolution of the parameters serum creatinin and bilirubin during MARS therapy. Each line represents a pair of values from an individual MARS treatment session. Gray dots represent values from uneventful MARS session, black open circles sessions complicated by device clotting and black triangles sessions with bleeding complications. Closed black circles connected by a thick black line indicate mean values. Significant *P* values are indicated, sessions analysed (*n* = 25), of which (*n* = 16) were uneventful, (*n* = 3) were complicated by bleeding and (*n* = 6) by clotting of the MARS filter system.

Serum concentrations of creatinin, bilirubin arterial ammonium and CRP were determined before and after each MARS session. As reported previously, serum creatinin and bilirubin were significantly cleared by MARS (Figure 2) [8]. Serum ammonia and CRP were unaffected by MARS (not shown).

### 3.3. Complications during MARS Treatment and Baseline Clotting Parameters

Complications due to MARS treatment occurred during 9 of the 25 sessions (36%). Bleeding from catheter insertion sites necessitating termination of the MARS sessions occurred during 3 sessions (patient no. 2, session 1; patient no. 4, session 6 and 7). MARS was definitively discontinued in patient no. 4. In patient no. 2, MARS therapy was resumed after one week and unfractionated heparin was omitted due to previous bleeding events; however, the two subsequent sessions (2 and 3) had to be stopped prematurely because of clotting within the MARS filter system. In addition, filter clotting lead to shortening of session 2 and 4 in patient no. 3. Filter clotting happened despite application of 400 IU/h of unfractionated heparin (patient no. 5) or of epoprostenol sodium (patient no. 3; Table 1).

### 3.4. Prediction of Complicated MARS Sessions

**Figure 3:**
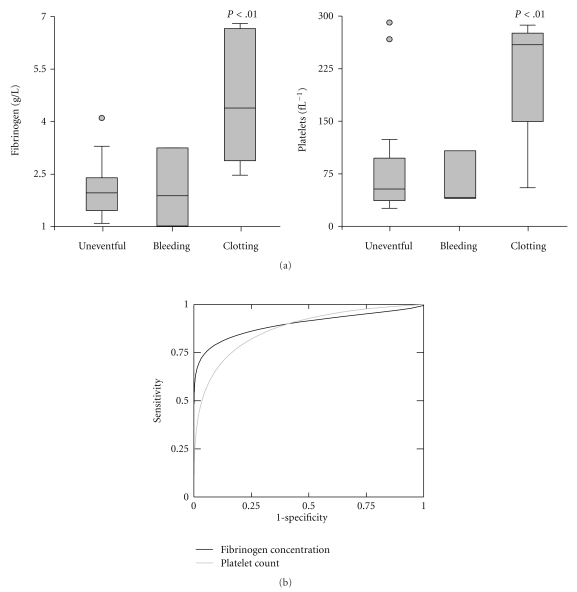
High plasma fibrinogen concentration and lack of thrombocytopenia at baseline predict clotting within the MARS filter system. (a) Baseline fibrinogen concentrations and platelet counts from individual sessions were grouped into sessions without event (uneventful), complicated by bleeding episodes and complicated by clotting events within the MARS filter system. (b) Receiver operator curve of baseline plasma fibrinogen concentration and platelet count for prediction of MARS filter clotting events. Significant *P* values are indicated, AUC_ROC_ = 0.94 (CI_95%_ 0.74 to 0.99) for fibrinogen, 0.82 (CI_95%_ 0.5 to 0.95) for platelets, respectively, sessions analysed (*n* = 25), of which (*n* = 16) were uneventful, (*n* = 3) were complicated by bleeding and (*n* = 6) by clotting of the MARS filter system.

In order to explore predictors for bleeding or clotting events during MARS, we compared various coagulation parameters prior to sessions with and without events. None of the parameters analysed at baseline (INR, aPTT, TT, fibrinogen content, D-dimer, platelet count, CT, CFT, MCF, or Lys 60′) were significantly associated with developing bleeding complications. In contrast, fibrinogen content and platelet counts were significantly higher prior to sessions that were later complicated by MARS filter clotting (Figure 3(a)). The predictive value of a parameter can be estimated by calculation of the area under the curve (AUC) in receiver operator curves (ROC). AUC_ROC_ were found to be 0.94 for fibrinogen and 0.82 for platelets (Figure 3(b)). In our cohort, a fibrinogen content of 2.5 g/L or more was found to predict filter clotting with 100% sensitivity and 80% specificity. Platelet counts exceeding 150 fL^−1^ still had a 80% sensitivity and a specificity of 80%.

### 3.5. Changes in Clotting Parameters during MARS Therapy

**Figure 4:**
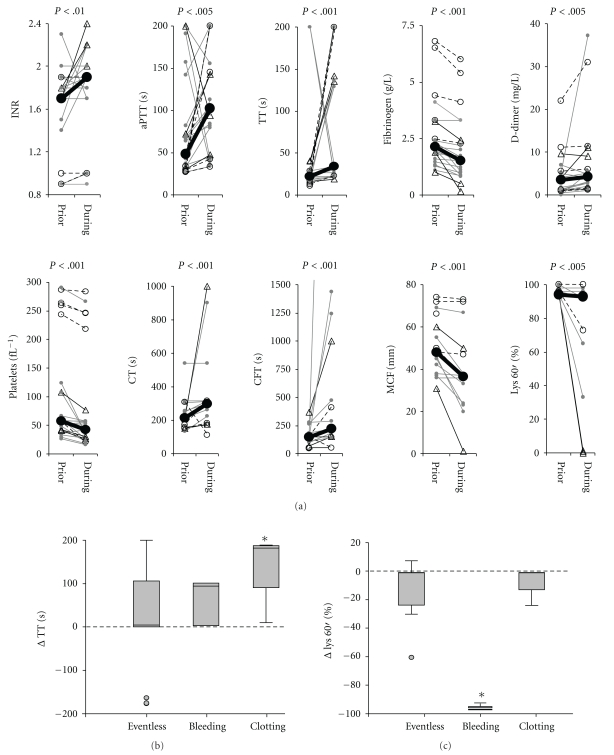
Clotting parameters deteriorate during MARS therapy: (a) Evolution of the clotting parameters INR, aPTT, TT, plasma fibrinogen concentration, D-Dimer, platelet count and the thromboelastography parameters CT, CFT, MCF and Lys 60′ during MARS therapy. Each line represents a pair of values from an individual MARS treatment session. Gray dots represent values from uneventful MARS session, black open circles sessions complicated by device clotting and black triangles sessions with bleeding complications. Closed black circles connected by a thick black line indicate mean values. All parameters worsened significantly during MARS sessions, *P* values are indicated, *n* = 16 to 25. (b) and (c) Sessions of MARS therapy were split up into 3 subgroups, uneventful sessions (eventless, *n* = 16), sessions with bleeding complications (bleeding, *n* = 3) and sessions complicated by device clotting (clotting, *n* = 6). For each individual session the value obtained at around 2 h of MARS therapy was subtracted from the baseline value. For each parameter, the differences were compared between subgroups by ANOVA and in case of significance Bonferroni group comparison was performed. ANOVA was only significant for the parameter TT (b) and Lys 60′ (c). *Indicates significantly different subgroups at the *P* < .05 level, *n* = 19 (b) and 13 (c), respectively.

INR, aPTT, TT, fibrinogen content, D-dimer, platelets and the thromboelastography parameter CT, CFT, MCF, and Lys 60′ were all found to significantly deteriorate around 2 hours of MARS therapy (Figure 4(a)). In most sessions the parameters either normalized spontaneously or due to transfusion of clotting substrates or platelets. The latter is the reason why the focus of our analysis is on the initial phase of MARS therapy.

To test if clotting parameters were differently affected by MARS in the subgroup of MARS sessions without complications, with bleeding complications and sessions complicated by clotting events ANOVA was performed. ANOVA was significant for changes in TT and hyperfibrinolysis (Lys 60′). Bonferroni pairwise comparisons revealed that the increase of TT during MARS was significant in the subgroup of MARS sessions complicated by device clotting events (Figure 4(b)). Device clotting occurred despite the usage of heparin (2 out of 6 sessions) or epoprostenol (2 out of 6 sessions; Table 1). The parameter indicating hyperfibrinolysis (Lys 60′) was significantly affected by MARS in sessions complicated by bleeding events (Figure 4(c)). For all other parameters (INR, aPTT, fibrinogen content, D-dimer, platelets CT, CFT, MCF and Lys 60′) no subgroup specific alteration by MARS could be detected.

### 3.6. Predicting Bleeding Complications by Monitoring Clotting Parameters

**Figure 5:**
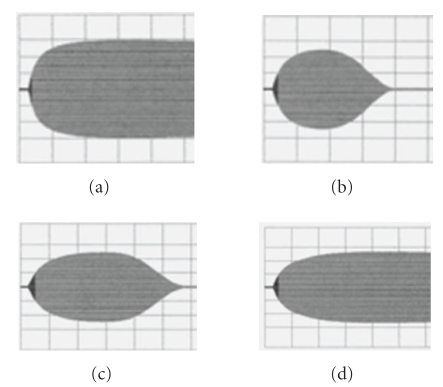
Thromboelastography detects fibrinolysis during MARS: Print outs of thromboelastography assessed during MARS session 1 in patient no. 2 which was complicated by bleeding after 2 hours of MARS therapy. Blood samples were obtained before (a) 1hour after (b) and 2 hours after (c) initiation of MARS. (d) shows thromboelastography one hour after MARS was stopped and substitution therapy was initiated. Grids and parameters are as explained in Figure 1.

We expected that hourly monitoring of clotting parameters during MARS therapy would identify patients at risk for bleeding complications. After one hour of the first MARS session in patient no. 2 thromboelastography revealed signs of fibrinolysis (Figure 5). In view of the yet unaffected parameters INR, aPTT, TT, fibrinogen content, D-dimer and platelet count MARS was not discontinued until when bleeding occurred two hours after starting MARS. The stopping of MARS treatment together with substitution of fibrinogen (2 g), fresh frozen plasma (2 units) and platelets (1 unit) and therapy with tranexamic acid (40 mg/kg body weight) bleeding stopped and clotting parameters improved reaching baseline values within one hour (Figure 5) of substitution. 

 In contrast, monitoring failed to announce the bleeding complications in patient no. 3. In this patient bleeding occurred during the sixth and the seventh MARS sessions. Baseline INR, aPTT, TT, fibrinogen content, D-dimer, platelet count and thromboelastography parameters were not found to significantly differ between the two complicated and the previous 5 uncomplicated sessions. Despite the close (hourly) monitoring during MARS therapy, we failed to anticipate bleeding in this patient because of defibrination (fibrinogen 0.15 g/L) and bleeding occurred faster then the first results obtained with our monitoring.

### 3.7. Prothrombin Activation and Thrombin Potential in MARS Therapy

**Figure 6:**
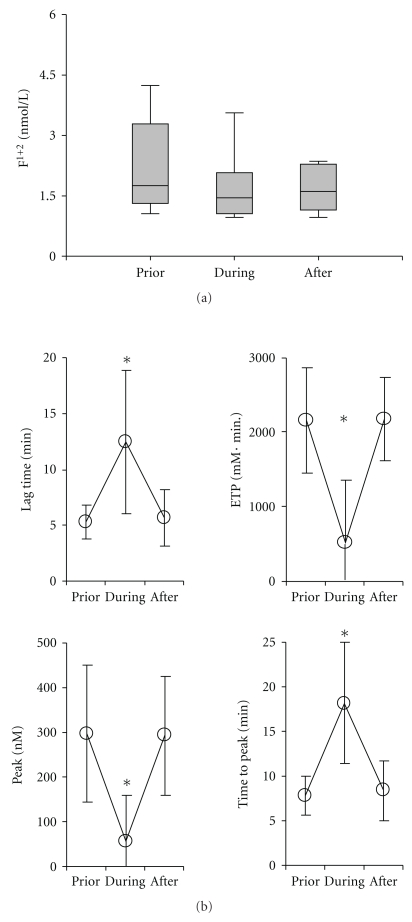
No indication for activation of the clotting system by MARS: From a subset of MARS sessions (patient no. 2 and no. 4) frozen plasma samples were available and parameters for thrombin activation were determined. (a) Quantification of F_1+2_ fragments by ELISA in plasma samples obtained before (prior) during and after the MARS sessions, are presented as box plots. Baseline F_1+2_ values were significantly above the cut-off value of 1.2 nmol/L (*P* < .05). MARS therapy did not significantly influence F_1+2_ values over time. (b) Estimation of the endogenous thrombin potential. Samples were as in (a). Line plots present data as mean ± SD for the parameters lag time, endogenous thrombin potential (ETP), maximal response (peak) and the time until the peak is reached (time to peak), *n* = 8, *Indicates *P* < .05.

To further explore the potential underlying mechanism driving clotting complications during MARS therapy we retrospectively quantified F_1+2_ peptide fragments. These activation peptides are generated upon the conversion of prothrombin into thrombin and serve as marker for thrombin generation. Baseline F_1+2_ were elevated and found to slightly decrease under MARS therapy (Figure 6(a)). 

Recently an old diagnostic parameter, the “thrombin potential” which correlates with coagulability and hence provides insight into the level of bleeding risk [16] has become relatively easy to determine. In a subset of the patients the thrombin potential was determined post hoc and was found to be significantly decreased during MARS in terms of timing (prolongation of lag time and time to peak) as well as in terms of response (decreased endogenous thrombin potential and decreased peak; Figure 5(b)). After MARS, the parameters recovered to baseline levels (Figure 6(b)).

## 4. Discussion

MARS treatment has been used in several thousand patients worldwide. Initially it was reported to be effective and safe and to improve surrogates such as bilirubin levels [8, 17–21], encephalopathy [17, 22], and as well as ameliorate systemic, renal and cerebral blood flow [17–19, 23–29]. Nevertheless, preliminary data from the yet unpublished RELIEF multi centre trial and meta-analysis [5] based on 4 randomized controlled trials [17–19] reported no survival benefit of MARS therapy. 

Could it be that MARS efficacy failed because undesired side effects jeopardized benefits? MARS therapy indeed has been reported to worsen hepatic coagulopathy and eventually cause bleeding complications [8–10]. Our actual study confirms and extends these previous observations by demonstrating clotting parameters such as INR, aPTT, TT, plasma fibrinogen concentration, and platelet count, as well as the functional parameters of thromboelastography deteriorate during MARS therapy. 

 Could baseline screening help to identify patients at risk? Similar to previous studies [8–10], we found that baseline clotting parameters tend to be more pathologic in patients developing bleeding complications. Age, INR and D-Dimer level at baseline have been reported to significantly correlate with bleeding complications during MARS [8], however, so far no parameter or set of parameters have helped to accurately identify patients at risk [8–10]. 

Herein we now addressed if patients at risk can be identified while MARS therapy is applied and if early intervention might prevent clinically relevant bleeding episodes. We found that bed-side thromboelastography reliably detected hyperfibrinolysis in all sessions with bleeding episodes. Given the small number of events (*n* = 3) we, however, could not calculate predictive values. Although bed-side thromboelastography is considered to rapidly generate parameters, in one out of two patients hyperfibrinolysis and severe defibrination occurred so fast, that is, within the first hour of MARS therapy, that fibrinolysis was only recognized by thromboelastography when bleeding had already occurred also clinically. Thromboelastography requires a clot to be formed in the first place to detect fibrinolysis. If substitution of InTeg activator by a more rapid agonist and complementation of the patient sample with factors such as fibrinogen could accelerate detection of hyperfibrinolysis and be more sensitive will have to be addressed in future studies. 

Instead of simply identifying patients prone to bleeding, targeting the underlying mechanism triggered by MARS would be the desired therapeutic option. We found fibrinolysis in both patients who suffered form bleeding complications, This suggests that plasminogen activation results in hyperfibrinolysis and would thus be the main reason for the bleeding complications. Uncontrolled plasminogen activation is a recognized complication of extra corporal devices involving foreign membranes [7]. Given the small study population we, however, cannot rule out other mechanisms to cause bleeding complications other than fibrinolysis as reported by other studies. We, therefore, would not recommend preemptive antifibrinolytic therapy for patients with severely altered clotting parameters undergoing MARS therapy. 

Besides hyperfibrinolysis underlying thrombocytopenia, platelet dysfunction, dysfibrinogenaemia, disseminated intravasal coagulation, reduced clearance of activated clotting factors and endothelial dysfunction, common in patients suffering from liver disease [6], certainly contribute to bleeding complications. Extra corporal blood flow, blood pumps, filter systems and bubble traps further aggravate such disorders [7] and decrease in clotting factor VIII and von Willebrand factor have been reported [9]. These findings support aggravation of disseminated intravasal coagulation. In contrast we found the F_1+2_ fragments, products released upon conversion of prothrombin, to decrease during MARS and the thrombin potential to decrease upon therapy. Although this observation would argue against aggravation of consumptive coagulopathy, clearance of F_1+2_ peptide by MARS which uses a 45 kD cut-off membrane and factor depletion triggered by consumptive coagulopathy could explain our findings. 

Whereas most alterations of clotting parameters must be attributed to the MARS therapy itself, some alterations might—at least in part—be explained by the application of low-dose heparin. The prolonged thrombin time reported in uncomplicated MARS sessions and sessions complicated by device clotting might be caused by heparin. 

Besides bleeding, device clotting complicates MARS therapy. We found that lack of thrombocytopenia and elevated plasma fibrinogen concentrations predict clotting events and that both platelet inhibition with epoprostenol and the administration of unfractionated heparin could not prevent filter clotting. If combining either anticoagulant or citrate anticoagulation (as is used routinely for hemofiltration) would prevent such events will have to be addressed in future research. 

 In conclusion, our study shows that bleeding complications are a major concern of MARS therapy. Extended baseline monitoring cannot accurately identify patients that will develop bleeding complications. However, we found that normal or increased platelet counts and elevated plasma fibrinogen concentrations at baseline allow predicting clotting of the MARS filter system. Although close monitoring of clotting parameters and especially monitoring by bed-side thromboelastography can anticipate bleeding complications, bleeding can still develop before clotting parameters become available.

## Figures and Tables

**Table 1:** Duration of individual MARS sessions, usage of anticoagulants and observed complications.

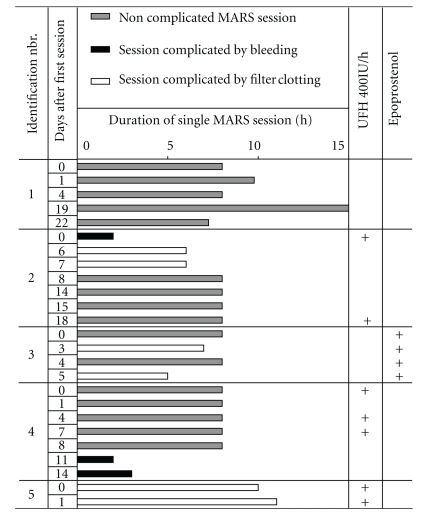

**Table 2:** Reference values of activated Thromboelastography.

Parameter	2.5 percentile	97.5 percentile	Median	(min./max.)
CT (seconds)	110.1	234.3	136	(103/257)
CFT (seconds)	42.2	128.8	74	(38/142)
MCF (mm)	47.1	67.0	58	(45/70)
Lys 60 (%)	98	89	96	(98/88)

**Table 3:** Demographics and laboratory data prior to MARS treatment.

Identification number	Gender (women/men)	Age (years)	Indication for MARS^1^	Number of MARS sessions performed	Child Pugh Points^6^	MELD Points^7^	SAPS II Points^8^	Number of Organs/Systems in Failure^9^	Serum-Bilirubin^10^ (*μ*mol/L)	Serum-Creatinin^11^ (*μ*mol/L)	Arterial Ammonia^12^ (*μ*mol/L)	INR^13^	Platelet count^14^ (×10^9^/L)
1	w	45	ALF^1^	5	10	13	69	3	306	56	102	1.7	60
2	w	50	AoCLF^2^	7	10	22	33	5	400	145	58	1.8	108
3	w	41	Pruritus^3^	4	5	6	7	0	7	70		1	291
4	m	63	HRS^4^	7	11	13	55	3	125	204	56	1.8	35
5	m	82	Intox^5^	2	6	6	61	3	4	97	12	1	264

median		**50**		**5**	**10**	**13**	**55**	**3**	**108**	**97**	**57**	**1.75**	**108**

^1^Acute liver failure, due to liver infiltration by lymphoma.

^2^Acute on chronic liver failure, the underlying liver disease was nonalcoholic steatohepatitis triggered by infection.

^3^Primary biliary cirrhosis with refractory pruritus.

^4^Hepatorenal syndrome due to Child-Pugh C cirrhosis attributable to alcoholic liver disease.

^5^Severe intoxication with clorazepate.

^6–8^According to the following publications: 6: to [12]; 7: to [13]; 8: to [14].

^9^Number of organs/systems requiring pharmacologic and/or mechanical support.

^10–14^Normal value/range: 10: <17 *μ*mol/L; 11: 70–150 *μ*mol/L; 12: <33 *μ*mol/L; 13: <1.2; 14: 143–400 × 10^9^/L.
